# Loud noise-exposure changes the firing frequency of subtypes of layer 5 pyramidal neurons and Martinotti cells in the mouse auditory cortex

**DOI:** 10.3389/fnagi.2023.1152497

**Published:** 2023-05-04

**Authors:** Ingrid Nogueira, Thiago Z. Lima, Thawann Malfatti, Katarina E. Leao

**Affiliations:** Hearing and Neuronal Activity Lab, The Brain Institute, Federal University of Rio Grande do Norte, Natal, Brazil

**Keywords:** auditory system, electrophysiology, tinnitus mechanisms, whole-cell patch clamp, principal component analysis

## Abstract

**Introduction:**

Loud noise-exposure can generate noise-induced tinnitus in both humans and animals. Imaging and *in vivo* studies show that noise exposure affects the auditory cortex; however, cellular mechanisms of tinnitus generation are unclear.

**Methods:**

Here we compare membrane properties of layer 5 (L5) pyramidal cells (PCs) and Martinotti cells expressing the cholinergic receptor nicotinic alpha 2 subunit gene (*Chrna2*) of the primary auditory cortex (A1) from control and noise-exposed (4–18 kHz, 90 dB, 1.5 h, followed by 1.5 h silence) 5–8 week old mice. PCs were furthermore classified in type A or type B based on electrophysiological membrane properties, and a logistic regression model predicting that afterhyperpolarization (AHP) and afterdepolarization (ADP) are sufficient to predict cell type, and these features are preserved after noise trauma.

**Results:**

One week after a loud noise-exposure no passive membrane properties of type A or B PCs were altered but principal component analysis showed greater separation between type A PCs from control and noise-exposed mice. When comparing individual firing properties, noise exposure differentially affected type A and B PC firing frequency in response to depolarizing current steps. Specifically, type A PCs decreased initial firing frequency in response to +200 pA steps (*p* = 0.020) as well as decreased steady state firing frequency (*p* = 0.050) while type B PCs, on the contrary, significantly increased steady state firing frequency (*p* = 0.048) in response to a + 150 pA step 1 week after noise exposure. In addition, L5 Martinotti cells showed a more hyperpolarized resting membrane potential (*p* = 0.04), higher rheobase (*p* = 0.008) and an increased initial (*p* = 8.5 × 10^–5^) and steady state firing frequency (*p* = 6.3 × 10^–5^) in slices from noise-exposed mice compared to control.

**Discussion:**

These results show that loud noise can cause distinct effects on type A and B L5 PCs and inhibitory Martinotti cells of the primary auditory cortex 1 week following noise exposure. As the L5 comprises PCs that send feedback to other areas, loud noise exposure appears to alter levels of activity of the descending and contralateral auditory system.

## Introduction

Several studies report adaptive changes in the primary auditory cortex (A1) following acoustic overstimulation of animals (Popelár et al., 1987; Seki and Eggermont, 2002; Noreña and Eggermont, 2003; Sun et al., 2008; Munguia et al., 2013; Basura et al., 2015; Takacs et al., 2017). For example, larger evoked potentials and increased firing frequency in the auditory cortex has been observed immediately following noise exposure of rats (Sun et al., 2012). Also, an increase in spontaneous firing frequency of the A1 has been shown 4–6 weeks following noise-induced tinnitus in guinea pigs (Basura et al., 2015). Recently noise overexposure was shown to increase the firing gain of A1 pyramidal neurons projecting to the inferior colliculus and areas of the limbic system (striatum and amygdala) for up to 2 weeks after the noise exposure (Asokan et al., 2018). Yet, whether loud noise can have persistent effects on pyramidal neurons with different projection profiles, or whether subtypes of pyramidal neurons are more vulnerable to noise exposure, is unknown.

Cortical pyramidal cells (PCs) are heterogeneous in respect to connectivity, as well as morphology and laminar distribution (Mason and Larkman, 1990; Harris and Shepherd, 2015; Wang et al., 2018). Especially layer five, constituting the main output neocortical layer, PC heterogeneity has been extensively studied (Molnár and Cheung, 2006; Morishima and Kawaguchi, 2006). Layer 5 PCs can be divided into many subtypes based on their specific projections but for simplification L5 PCs are often grouped into two main subtypes: corticofugal and commissural-projecting PCs (Oswald et al., 2013). We have previously used the distinction of type A and type B L5 PCs based on electrophysiological profile, where type A PCs correspond to large L5 PCs with thick-tufted dendrites, to study connectivity between L5 PCs and inhibitory Martinotti cells (Hilscher et al., 2017). Moreover, type A are known to express prominent h-current (Ih), AHP, ADP, and project subcortically, while type B PCs are thin-tufted, have little Ih, AHP, ADP, and connect contralaterally or to the striatum (Lee et al., 2014; Hilscher et al., 2017). Another study by Joshi et al. (2015) retrogradely labeled PCs in the A1 from inferior colliculus and contralateral A1 and could also distinguish two different types of PCs with different hyperpolarization-induced sag amplitude (indicating Ih) taking part of two functionally distinct synaptic pathways of input-output of the A1. In addition we have previously shown layer 5/6 inhibitory Chrna2 positive (+) Martinotti cells to preferentially connect to apical dendrites of type A PCs and generate rebound excitation important for type A PC synchronization (Hilscher et al., 2017). Still, how excessive loud noise affects membrane properties of different L5 PCs and specific interneurons of the A1 is still not clear. Here we compare whole-cell patch clamp recordings and principal component analysis of L5 type A and type B PCs, and genetically defined inhibitory Chrna2-Martinotti cells of the A1 from control and noise-exposed mice. We found that loud noise, which can cause acute noise-induced tinnitus (Winne et al., 2020; Malfatti et al., 2022), alters the firing frequency of L5 main cell types 1 week after the noise overexposure.

## Materials and methods

### Animals

A total of 19 wild type mice (c57BL/6) of either sex, age between 2 and 3 weeks (*n* = 4) and 5–8 weeks (*n* = 15) and 5–8 weeks (*n* = 10) Chrna2-cre mice bred with homozygote tdTomato reporter mice on a mixed genetic background (Sv129:c57BL/6) (Madisen et al., 2010; Leão et al., 2012) of either sex were used in this study. All experimental procedures followed current guidelines and were approved by the Ethics Committee for the Use of Animals of Federal University of Rio Grande do Norte (CEUA/UFRN) protocol no 097.019/2018 and 135.064/2018. Animals were housed on a 12/12 h day/night cycle and had free access to food and water.

### Noise exposure

Acoustic noise overexposure was carried out in a sound shielded room, inside a sound-isolated cabinet (44 cm × 33 cm × 24 cm) during the late afternoon. Mice were handled and habituated for 3–5 days by being placed inside an acrylic cylinder (diameter 4 cm × 8 cm length), with restraining doors perforated at regular intervals (Acrilart, Natal-RN, Brazil), for 5–10 min at a time. Mice were considered habituated when freely entering the cylinder and there was minimal trace of defecation. A speaker (Selenium Trio ST400) connected to a sound amplifier (Marantz PM8004) and sound board (USBPre2), was placed 10 cm in front of the acrylic cylinder to produce the sound stimulation. The speaker was calibrated using a microphone (Brüel and Kjaer 4939-A-011) and adjustment of intensity, frequency and duration of the sound was done using custom written code (Matlab, MathWorks, Natick, MA, USA). Awake mice were exposed to broadband noise of 4–18 kHz, at 90 decibel sound pressure level (dBSPL) for 1.5 h (Winne et al., 2020) to over-activate a large portion of the auditory cortex. Immediately following noise overexposure animals were removed from the acrylic cylinder but remained in the sound shielded cabinet, inside a standard plastic cage for another 1.5 h in silence. This was done since increased ambient noise and acoustic enrichment immediately following a noise trauma can prevent noise-induced tinnitus (Sturm et al., 2017). Following the silence period animals were returned to their home cages in the animal facility for 1 week before being sacrificed for electrophysiological experiments. Control animals were age matched littermates. We have previously shown a noise level of 90dBSPL, followed by a brief period of silence, to cause significant impairment in gap pre-pulse inhibition of acoustic startle (30/34 mice showed decreased gap detection capabilities for at least two frequency bands tested) indicating acute noise-induced tinnitus (Malfatti et al., 2022).

### Whole-cell patch clamp

Young mice (P16-23) were sacrificed by decapitation and thereafter immediate brain dissection. To improve cell visibility and cell survival of slices from more mature mice (>5 weeks old), mice were routinely perfused prior to slicing and had recovery solution applied (Ting et al., 2014). In detail, mature animals (P38-52) were sacrificed by intraperitoneal injection with ketamine (90 mg/kg) for anesthesia before intracardiac perfusion with artificial cerebrospinal fluid (ACSF) containing (in mM): NaCl, 124; KCl, 3.5; NaH_2_PO_4_, 1.25; MgCl_2_, 1.5; CaCl_2_, 1.5; NaHCO_3_, 30; glucose, 10. Brains were rapidly dissected, the cerebellum and brainstem removed, glued to a platform and submerged in ice-cold sucrose/artificial cerebrospinal fluid (ACSF) consisting of the following (in mM): KCl, 2.49; NaH_2_PO_4_, 1.43; NaHCO_3_, 26; glucose, 10; sucrose, 252; CaCl_2_, 1; MgCl_2_, 4. The brain was cut in coronal slices (300 μm thick) using a vibratome (VT1200, Leica, Microsystems) and slices containing the A1 were collected and moved to a holding chamber containing normal ASCF, or for >1 month old mice containing *N*-methyl-D-glucamine (NMDG, recovery) solution (in mM): NMDG, 93; KCl, 2.5; NaH_2_PO_4_, 1.2; NaHCO_3_, 30; HEPES, 20; sodium ascorbate, 5; thiourea, 2; sodium pyruvate, 3; hydrated MgSO_4_; 10; CaCl_2_, 0,5, pH calibrated with HCl to pH 7.3–7.4, for 12 min to improve cell survival and cell visibility in *in vitro* slices (Ting et al., 2014), and next being placed in normal ACSF constantly bubbled with 95% O_2_ and 5% CO_2_ at room temperature (22–24°C). Next slices were transferred to a submerged chamber under an upright microscope equipped with differential interference contrast (DIC) optics (Olympus, Japan) and perfused with room temperature oxygenated ASCF (1–1.25 ml/min). Patch pipettes from borosilicate glass capillaries (GC150F-10, Harvard Apparatus, MA, USA) were pulled on a vertical puller (PC-10, Narishige, Japan). Pipette resistances varied from 8 to 12 MΩ. Pipettes were filled with internal solution containing (in mM): K-gluconate, 130; NaCl, 7; MgCl_2_, 2; ATP, 2; GTP, 0.5; HEPES, 10; EGTA, 0.1 (from Sigma Aldrich, MO, USA). The pH was adjusted to 7.2 using KOH. Whole-cell current clamp recordings were acquired using an Axopatch 200B amplifier (Axon instruments, CA, USA) and digitized with a BNC-2111 panel block (National instruments, TX, USA). The primary auditory cortex was identified using the mouse brain atlas (Paxinos and Franklin, 2012) by recognizing the shape of the hippocampus and the rhinal fissure in coronal sections, and next consistently patching from layer 5 (identified visually based on the internal edge of the cortex, tissue density and cell morphologies). Pyramidal cells were identified by size and morphology and routinely clamped to −65 mV before breaking in. The cholinergic receptor nicotinic alpha 2 subunit-positive (*Chrna2*+) Martinotti cells (Hilscher et al., 2017) of layer 5/6 of the A1 were identified by red fluorescent protein in brain slices from Chrna2-cre/tdTomato-lox mice. WinWCP software implemented by Dr. J. Dempster (University of Strathclyde, Glasgow, UK) was used to record electrophysiological signals. Cells with an unstable baseline, membrane resistance and/or more depolarized resting membrane potential than −50 mV (pyramidal cells) or −45 mV (Martinotti cells) were discarded from further analysis.

### Data analysis

Matlab (version 2016a, MathWorks) was used for data analysis of recordings. Resting membrane potential (Vrest) was noted as the baseline in current clamp mode. Membrane resistance was calculated from the current activated by a small test step (5 mV, 10 ms). Rheobase is the minimum amount of current necessary to generate an action potential (calculated from a ramp protocol from 0 to 200 pA, 500 ms, where the time of the first spike was noted; AP time). Hyperpolarizing sag amplitude was quantified in response to a negative current steps (−100 pA, 500 ms) as the difference between peak and steady-state voltage (ΔV mV). The afterdepolarization (ADP) and afterhyperpolarization (AHP) were measured following the termination of a −100 pA or + 150 pA step (500 ms duration) respectively, as the peak amplitude subtracted by Vrest. The first AP generated upon positive current injections (ramp from 0 to 200 pA, 500 ms) was analyzed for AP threshold (>10 mV/ms). We also examined the properties of APs using phase-plane plots, which show the derivative of membrane potential (dVm/dt) as a function of instantaneous membrane potential. Phase plots were obtained by plotting dV_m (obtained using the matlab command *diff*. vs. V_m.top). Firing frequency was analyzed from AP generated by depolarizing current injections (50 to 400 pA, 50 pA increments, 1 s duration). Initial frequency denotes the frequency of the first two APs, calculated as the inverse of the first interspike interval (ISI). Steady-state frequency denotes the frequency of the last 3 APs, calculated as the inverse of the mean of the last three interspike intervals. The initial and steady-state gain was calculated by fitting a trendline to both initial and steady state frequency in response to 150, 200, and 250 pA current steps and quantifying the slope (Hz/pA). For Martinotti cells the current clamp steps applied were in either 20 pA or 50 pA increments (−40 to 150 pA, or −100 to 400 pA, respectively, 500 ms or 1 s duration).

### Statistical analysis

For statistical analysis of the predictability of cell type and effect of condition, the experiment followed a 2^2^ factorial design, hence with 2 factors: cell type and noise-exposure (experimental condition), both with 2 levels. Sample sizes (after outlier removal) were 11 (8), 13 (8), 19 (17), and 21 (19) for groups A-control, B-control, A-noise, and B-noise, respectively. Principal component analysis (PCA) was computed from the correlation matrix of quantitative variables of the dataset, namely: absolute sag, ADP and AHP, resting potential, input resistance, AP threshold, AP time, rheobase, initial ISI, initial frequency, steady-state ISI and frequency, initial and steady state frequency-current gain. The principal components (PrC) were derived from the correlation matrix as the successive pairs of eigenvectors-eigenvalues. For each PrC, corresponding eigenvalue reflects the amount of information accounted for by it. In turn, each eigenvector indicates the coefficients for the linear combination of original variables that represents, in geometrical terms, the rotation toward the directions of the correlation matrix with highest variability. Thus, the meaning of each PrC was interpreted according to the signal and absolute value of these coefficients that generated it. The model for cell type classification was developed by means of logistic regression. When separated according to cell type only, all variables were shown to be compatible with the normal distribution. The selection of variables to the model was assisted by forward and backward stepwise selection and highly correlated variables were avoided. The model was defined based on the lowest deviance and AIC (Akaike information criteria) obtained. To perform parametric inference, 8 outliers were discarded because they were hampering the normalization of at least one variable. The variables incompatible with the normal distribution were transformed by injective functions: Box-Cox transform, Ln or inverse. Then, the effect of factors was tested by multivariate analysis of variance (MANOVA). To further identify response variables that most contribute for each effect, individual 2-way ANOVA was used as *post hoc*. For those untransformed variables, we also estimated effect sizes and an empirical model, which was based on multiple linear regression. For both ANOVA and linear regression, normality, independence, and variance homogeneity assumptions were checked by the analysis of the residues. Main effects were computed as the mean difference between levels of correspondent factor, whereas interaction effect was estimated by half the difference of the effect of one factor relative to both levels of the other factor. For all hypothesis tests, a significance level of 0.05 was used. For basic comparison between type A and type B variables and Martinotti cells from control and noise exposed mice shown in tables, two-tailed Student’s *t*-test, equal variance was applied, and data reported as standard error of the mean (s.e.m).

## Results

### Noise overexposure does not alter passive membrane properties important for classifying L5 PC type

**FIGURE 1:**
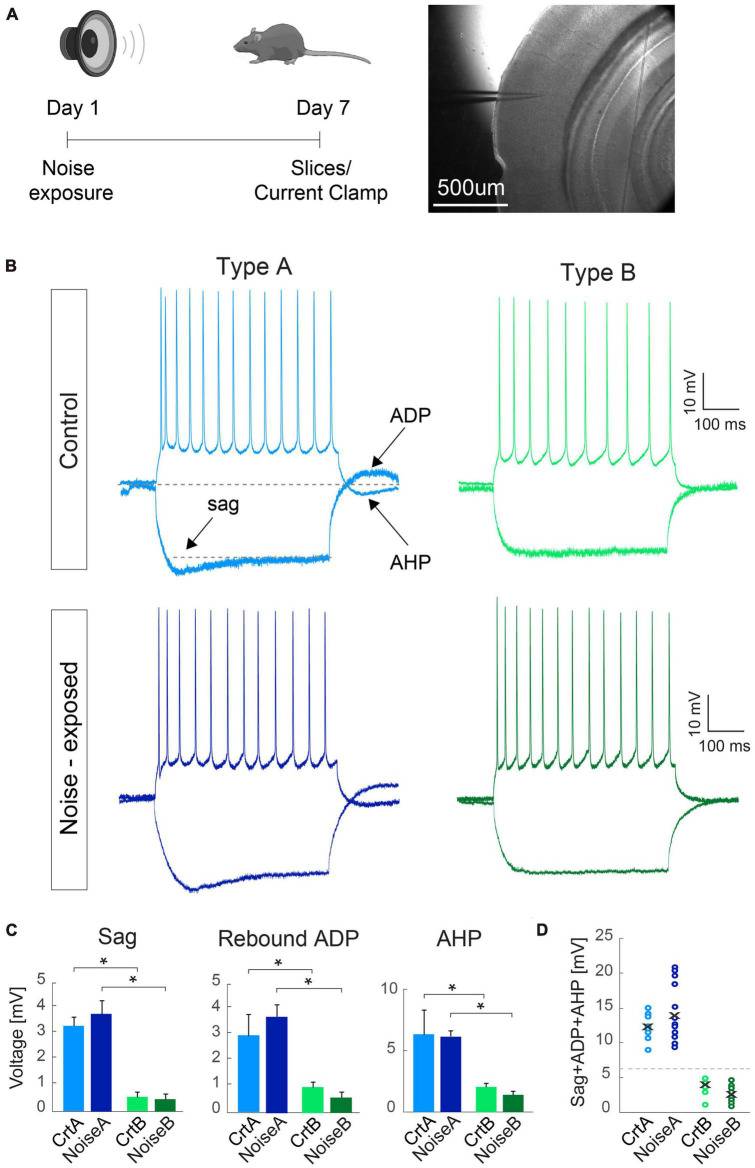
Criteria for separating L5 PCs into type A and type B remains robust between control and noise-exposed experimental groups. **(A)** Experimental schematic representation, Right: brightfield image of the primary auditory cortex with the pipette pointing toward layer 5. **(B)** Representative traces in response to –100 and 150 pA steps from L5 type A and type B PCs from control (top) and noise-exposed (bottom) mice. **(C)** Type A and type B cells show distinct values for hyperpolarizing sag, rebound afterdepolarization (ADP) and afterhyperpolarization (AHP) potential for control and noise-exposed groups. **(D)** Sum of sag, ADP and AHP show difference between type A and type B PCs from control (Crt) and noise-exposed (noise) mice. Error bars–s.e.m., Student’s *t*-test, two tailed, and equal variances, *indicates *p* < 0.05.

To test whether noise exposure (4–18 kHz at 90 dBSPL for 1.5, 1.5 h silence post noise exposure) affects L5 PCs firing properties, we performed current clamp recordings of pyramidal cells (*n* = 87 cells, on average 5 cells per animal) in layer 5 of the primary auditory cortex 1 week following noise exposure (Figure 1A). Layer 5 PC main subtypes were identified *post hoc* by fitting a model for cell type classification based on logistic regression (Table 1) showing that afterdepolarization (ADP) and afterhyperpolarization (AHP) magnitude was sufficient for cell classification (maximum predictive power of the model) (Gee et al., 2012; Lee et al., 2014; Joshi et al., 2015; Hilscher et al., 2017). Thereby, L5 PCs are hereafter referred to as type A or type B PCs (Lee et al., 2014). Type A and type B PC are also possible to distinguish in slices from young mice (2–3 weeks old), however, membrane properties still develop in the first weeks of age (Supplementary Figure 1 and Supplementary Table 1). Thereby we only compare mature membrane properties between control and noise-exposed mice >5 weeks old (*n* = 64 cells).

**TABLE 1:** Logistic regression model for L5 pyramidal cell type classification.

Model		$\text{P}\,\left("\,\text{A}\,\text{cell}\,"\right)=\frac{e^{300.16+89.98\,A\,b\,s.A\,D\,P+43.68\,A\,b\,s.A\,H\,P}}{1+e^{-300.16+89.98\,A\,b\,s.A\,D\,P+43.68\,A\,b\,s.A\,H\,P}}$	
AIC	6	Predictive power = 1 (accuracy: 64/64)	
		Deviance	Degrees of freedom
Null		88.473	63
Residual		2.712610^−10^	61

AIC, akaike information criteria; Abs.ADP, absolute afterdepolarization, Abs.AHP, absolute afterhyperpolarization; P (“A cell”), probability of a cell to be classified as type A.

Examining each electrophysiological parameter separately we first show that noise exposure did not alter hyperpolarization sag, ADP and AHP in responses to a negative (−100 pA, 500 ms) and a positive (150 pA, 500 ms) current step for L5 type A or type B PCs from noise-exposed or control mice (Figure 1). In both conditions, type A PCs (*n* = 30) had pronounced sag, ADP and AHP compared to type B PCs (*n* = 34) that showed generally flat response following negative and positive current injections (Figure 1B). Differences between type A and type B PCs were equally recognizable following noise exposure (Figure 1B) with a >6 mV cut-off criteria of the sum of sag, ADP and AHP amplitude (Figures 1C, D), similar to previously shown (Lee et al., 2014).

Passive and active membrane properties of L5 PCs from control and noise exposed mice (Table 2) showed that type B PCs have a more hyperpolarized membrane potential than type A PCs but that resting membrane potential and input resistance are not altered for type A and type B PCs following noise exposure. Examining action potential properties showed no difference in action potential (AP) threshold, although type A PCs showed a trend toward a slightly depolarized AP threshold in cells from noise-exposed animals (ctr A: −46.8 ± 1.8 mV vs. noise A: −42.8 ± 1.2 mV, *p* = 0.068) while AP threshold was robust for type B PCs from the two groups (ctr B: −41.5 ± 1.9 mV vs. noise B: −42.2 ± 1.7 mV, *p* = 0.924).

**TABLE 2:** Type A and type B L5 pyramidal cells of the A1 show different firing frequencies in slices from noise-exposed animals compared to control mice.

	Type A PCs			Type B PCs		
	**Control**	**Noise exp.**	***p*-value**	**Control**	**Noise exp.**	***p*-value**
**P (days)**	**47.5 ± 1.2**	**43.6 ± 0.8**		**49.5 ± 1.1**	**44.7 ± 0.7**	
V_rest_ (mV)	-65.7 ± 1.5	-64.6 ± 1.5	0.630	-72.3 ± 1.4	-71.1 ± 1.2	0.514
R_inp_ (MΩ)	201.9 ± 25.4	201.2 ± 14.7	0.978	182.5 ± 11.1	221.4 ± 20.2	0.392
Rheobase (pA)	68.0 ± 4.3	70.0 ± 4.8	0.779	108.4 ± 9.5	110.0 ± 8.2	0.796
ΔSag (mV)	3.4 ± 0.3	3.8 ± 0.5	0.523	0.5 ± 0.1	0.5 ± 0.1	0.742
ΔADP (mV)	3.2 ± 0.9	3.9 ± 0.4	0.450	1.0 ± 0.1	0.6 ± 0.1	0.065
ΔAHP (mV)	6.3 ± 2.0	6.1 ± 0.4	0.902	2.0 ± 0.3	1.4 ± 0.2	0.096
AP_thres_ (mV)	-46.8 ± 1.8	-42.8 ± 1.2	0.068	-41.5 ± 1.9	-42.2 ± 1.7	0.924
f_ini_ at 150 pA (Hz)	64.9 ± 10.9	44.8 ± 5.4	0.075	33.2 ± 3.8	54.3 ± 8.1	0.052
f_ss_ at 150 pA (Hz)	20.3 ± 1.8	16.1 ± 1.2	0.050*	13.3 ± 1.3	19.5 ± 2.4	0.048*
f−I ini gain (Hz/pA)	0.46 ± 0.08	0.32 ± 0.03	0.055	0.38 ± 0.04	0.41 ± 0.09	0.782
f−I ss gain (Hz/pA)	0.09 ± 0.04	0.04 ± 0.003	0.143	0.07 ± 0.01	0.09 ± 0.02	0.460

Note that frequency is shown only in response to a 150 pA step. P, postnatal; Vrest, resting membrane potential; R_inp, input resistance; Rheobase, minimal current to cause an action potential; Sag, hyperpolarization sag; ADP, afterdepolarization; AHP, afterhyperpolarization; AP_thres, action potential threshold; f, firing frequency; ini, initial; ss, steady state; f-I gain, frequency over current gain; Student’s *t*-Test, two-tailed, and equal variance. Data show standard error of the mean (s.e.m). *p ≤ 0.05*.

**FIGURE 2:**
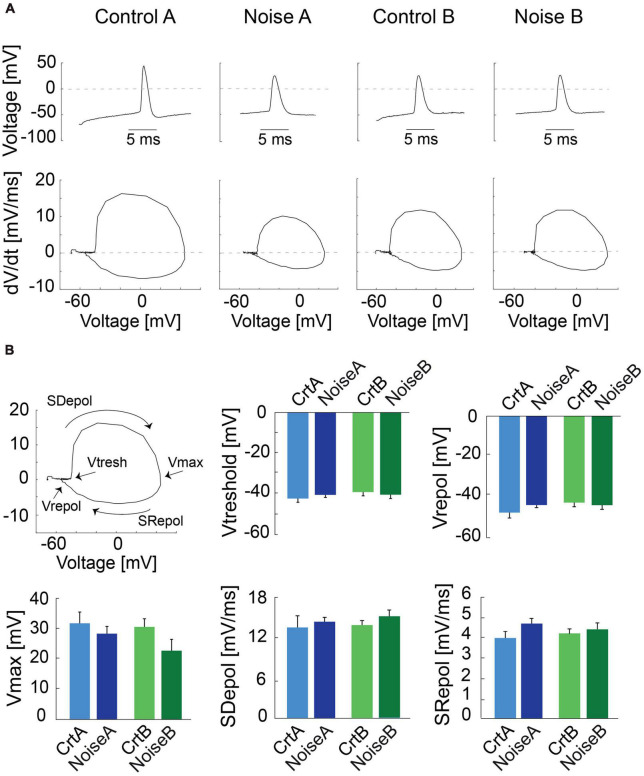
Phase plot analysis of action potentials from L5 PCs. **(A)** Representative traces of first APs recorded from control and noise-overexposed type A and B PCs in response to a 150 pA current injection (top) and the relative phase plots (bottom) showing depolarization and repolarization phases. **(B)** The first panel shows a phase plot and the representation of the threshold membrane potential (Vtresh), the maximal voltage peak of the AP (Vmax), the repolarization potential (Vrepol), and the upper and lower portions of the loop which represents the depolarization and repolarization phases (Slopes), respectively. Bar graphs of Vtreshold, Vmax, Vrepol, Sdepol and Srepol for type A and type B PCs from control and noise-exposed mice. Error bars–s.e.m.

To further investigate action potential (AP) shape we carried out phase plot analysis (Trombin et al., 2011) for control and noise-overexposed type A and type B L5 PCs (Figure 2A). The first derivative of the AP is represented as a loop highlighting the threshold membrane potential (*V*_*thres*_), and the maximal voltage amplitude (*V*_*max*_), with the depolarization and repolarization phases (slopes) characterized as the upper and lower portions of the loop, respectively (Figure 2B, left). Still, the phase plots did not reveal any differences of type A or type B PCs following noise exposure (Figure 2B). The slope of repolarization (S_*repol*_; ctr A: 3.9 ± 0.3 mV/ms vs. noise A: 4.7 ± 0.25 mV/ms, *p* = 0.09, and ctr B: 4.3 ± 0.2 mV/ms vs. noise B: 4.4 ± 0.3 mV/ms, *p* = 0.63) was not different following noise exposure. Neither was slope depolarization (S_*depol*_; ctr A: 1.35 ± 0.2 mV/ms vs. noise A: 1.4 ± 0.08 mV/ms, *p* = 0.66, and ctr B: 1.4 ± 0.08 mV/ms vs. noise B: 1.5 ± 0.09 mV/ms, *p* = 0.29) or Vmax and repolarization voltage (Figure 2B) showing that previous noise exposure does not alter the shape of the first action potential of L5 type A and type B PCs.

**FIGURE 3:**
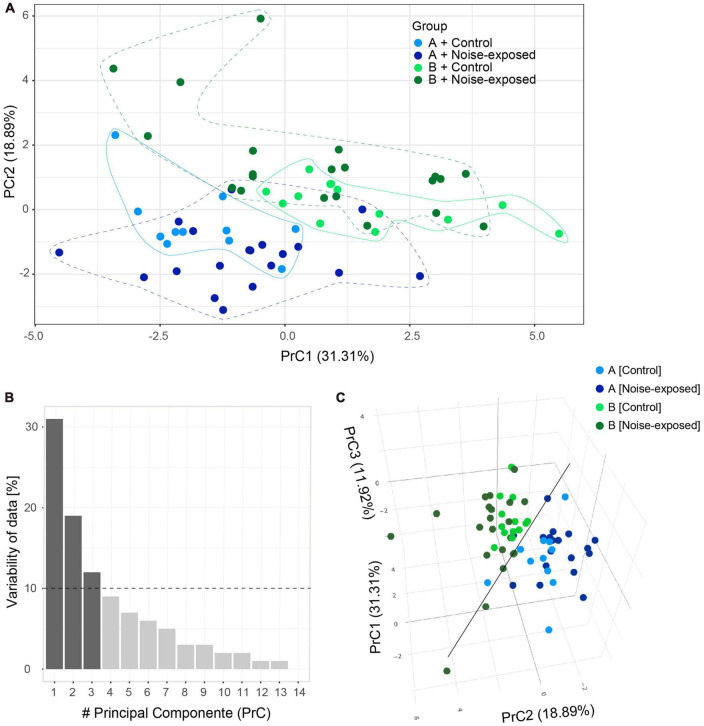
Principal component (PrC) analysis of PC parameters. **(A)** Visual clusterization of the data based on the first two PrC accounting for 50.2% of whole dataset information. Type A control (light blue), type B control (light green), type A noise-exposed (dark blue) and type B noise-exposed (dark green). Clusters of distribution of cells are highlighted with lines contouring the corresponding data points. **(B)** The relevance of each PrC, by the proportion of the whole dataset information relating to each PrC. The dotted line highlights the 10% cut-off for useful PrCs. **(C)** The inclusion of the third PrC adds 11.92% of whole dataset information to the visual clusterization. The black line highlights the separation of type B (upper in the plot) and type A (lower in the plot) pyramidal cells.

Next, principal component analysis based on the 11 electrophysiological parameters collected (Table 2) from control and noise exposed mice was carried out to illustrate any variability in the data from type A and type B PCs from control and noise exposed mice (Figure 3). The values of the first two principal components (PrC1-2, Supplementary Table 2) corresponded to 50% of the dataset (Table 2) information (i.e., variability, PrC1: 31%, PrC2: 19%, Figures 3A, B). Plotting the first and second principal components showed a minimal overlap between the type A and type B PC clusters, where type B cells (exhibited in dark colors) gather at greater values of the PrC2 than type A cells (Figure 3A). The first PrC was mostly relevant for separating cell types in control condition (Figure 3A). Addition of the third PrC (accounting for an additional 12% of variability, Figure 3B) allowed for a more comprehensive representation of the data in a 3D plot preserving 62% of the whole dataset information (Figure 3C). In the 3D plot the distinction between subtypes of PCs is visible (black line) and for type A PCs the experimental condition becomes more evident (Figure 3C). For type B cells, control and experimental conditions remain overlapped, but noise exposed type B PCs spread more than the control type B PCs cluster (Figure 3C). A multivariate analysis of variance (MANOVA) test confirmed that cell type had a significant effect (*p* = 1.58 × 10^–11^) on response variables and such effect was different in control and noise-exposed animals (Supplementary Table 3). This shows that type A and type B PCs are different electrophysiologically, but more importantly, that noise exposure increases variability but in different directions for type A and type B PCs.

### Noise overexposure alters firing frequency of type A and type B PCs in opposite direction

**FIGURE 4:**
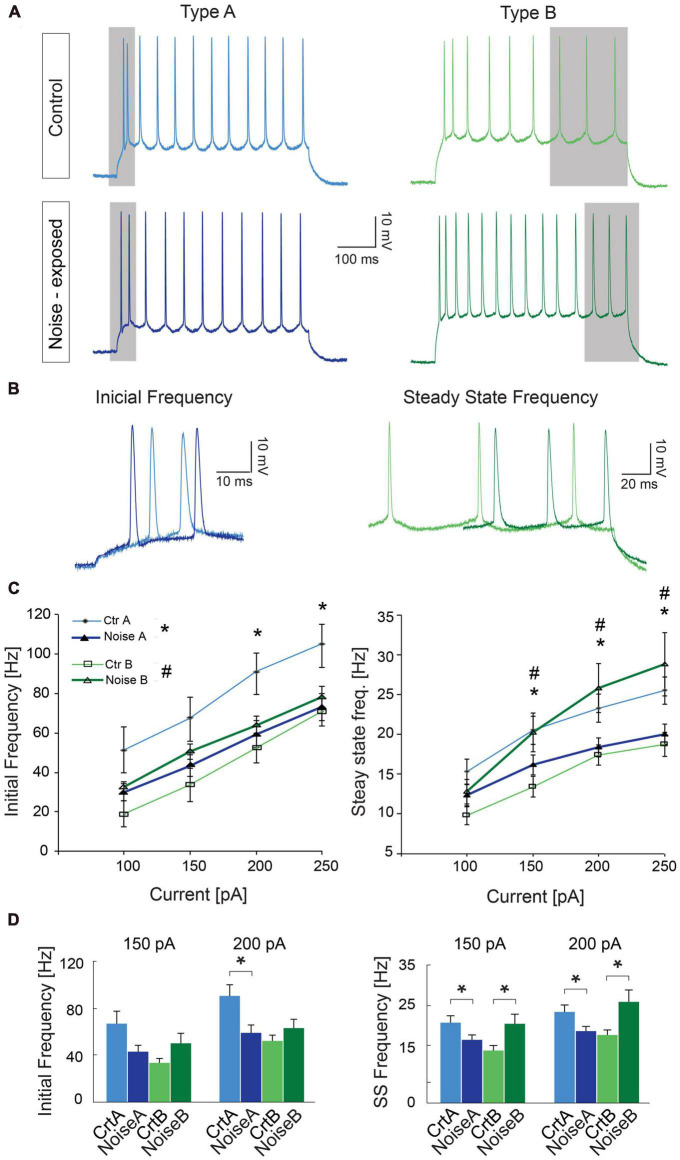
Noise exposure alters steady state frequency in opposite directions for L5 type A and type B PCs. **(A)** Representative traces illustrating firing frequency in response to a 1 s current injection of 150 pA. Gray shadows highlight the first two APs used for calculating initial frequency and the last 3 APs used for calculating steady-state frequency. **(B)** Higher magnification of traces highlighting the difference in initial firing frequency of L5 type A PCs from control and noise exposed mice (left), and the difference in steady state firing for control and noise-exposed L5 type B PCs. **(C)** Initial frequency over current plots shows a decrease in initial frequency for type A PCs after the noise overexposure (left). Right: steady state frequency-current plot shows significant increase in steady state frequency for type B PCs after noise exposure while type A cells on the contrary shows a decrease in steady state frequency. **(D)** Bar graphs showing initial and steady state firing frequency in response to a 150 and 200 pA stimulation. Error bars–s.e.m., Student’s *t*-test, two tailed, and equal variances. (*) denotes *p* < 0.05 for type A control vs. type A noise-exposure, (^#^) denotes *p* ≤ 0.05 for type B control vs. type B noise-exposure (see Table 2 for specific values).

As one of the main physiological outputs of electrophysiological membrane properties is regulation of firing frequency, responses to depolarizing current steps (100–250 pA, 1 s duration) was examined for type A and type B L5 PCs from control and noise exposed mice (Figures 4A, B). We found that the average initial firing frequency (from the interspike interval of the first two APs) was significantly lower for type A PCs 1 week after noise exposure in response to +200 pA (ctr A: 87.9 ± 9.6 Hz, *n* = 11 vs. noise A: 59.4 ± 6.8, *n* = 19, Hz, *p* = 0.02) and +250 pA (ctr A: 103.7 ± 9.8 Hz, *n* = 11 vs. noise A: 73.1 ± 6.8, *n* = 19, Hz, *p* = 0.014) current steps (Figures 4A–D). Type B PCs showed no difference in initial firing frequency in slices from control or noise exposed mice (Figure 4). The initial frequency increased linearly in response to 1 s duration current steps (100–250 pA) for both type A and type B PCs from both experimental conditions (Figure 4C, left). Noise exposure thereby appears to decrease the initial firing frequency of type A PCs and make initial frequency more similar to type B PC initial frequency (Figure 4C). Next, comparing steady-state firing frequency (last three APs in response to+150 pA step, 1 s duration) also showed a lower frequency for type A PCs from noise exposed mice (ctr A: 20.3 ± 1.8 Hz, *n* = 11 vs. noise A: 16.1 ± 1.2 Hz, *n* = 19, Hz, *p* = 0.050). On the contrary, type B PCs showed a higher average steady state firing frequency from noise exposed mice (ctr B: 13.3 ± 1.3 Hz, *n* = 13 vs. noise B: 19.5 ± 2.4 Hz, *n* = 21, *p* = 0.048, Figures 4A–D). Specifically, type B PCs from noise-exposed animals showed a higher firing frequency in response to increasing current injections (150–250 pA) compared to type A PCs and control type B PCs (Figure 4C, right).

**FIGURE 5:**
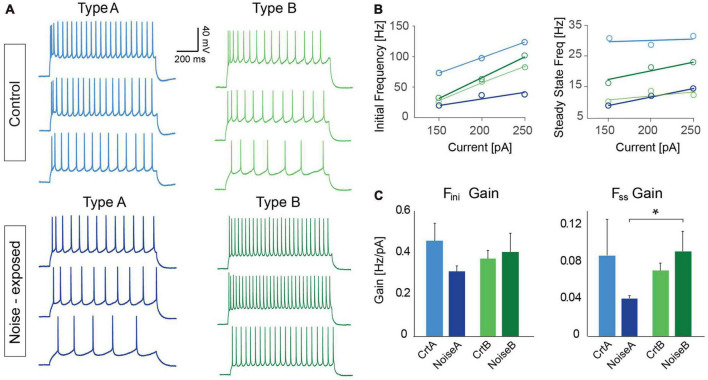
Frequency over current gain becomes significantly different between type A and type B PCs following noise exposure. **(A)** Representative current clamp traces in response to 150, 200, and 250 pA current injections (1 s duration) for type A and type B PCs from control (top) and noise-exposed (bottom) mice. **(B)** Example of a f-I plot showing initial frequency vs. current injection (left) and steady state frequency vs. current injection (right) for the neurons shown in **(A)**. **(C)** Bar graphs of gain (frequency/current) of initial firing frequency (left) and steady-state frequency (right) for L5 type A and type B PCs from control and noise-exposed mice. Error bars–s.e.m., Student’s *t*-test, two tailed, and equal variances, *indicates *p* < 0.05.

To further investigate differences in firing frequency we examined frequency-current (f-I) gain by comparing the average slope (Hz/pA) of initial and steady-state frequency in response to depolarizing current steps (150, 200, and 250 pA) of type A and type B L5 PCs (Figures 5A, B). Comparing the average slope of initial frequency showed a trend of type A PCs decreasing gain following noise exposure (ctr A: 0.46 ± 0.08 Hz/pA vs. noise A: 0.32 ± 0.03 Hz/pA, *p* = 0.055) while type B PCs showed no difference in initial firing frequency gain. For the average steady state frequency gain there was no difference between control and noise-exposed cells for type A or type B PCs (Figure 5C and Table 2). However, the differences in steady state gain becomes significantly different between type A and type B PCs following noise exposure (noise A: 0.04 ± 0.0 Hz/pA vs. noise B: 0.09 ± 0.02 Hz/pA, *p* = 0.03, Figure 5C, right). This supports that noise-exposure has persistent effects on firing frequency of type A and B PCs in the opposite direction.

### Chrna2-Martinotti cells fire at higher frequency in noise-exposed mice compared to control

**FIGURE 6:**
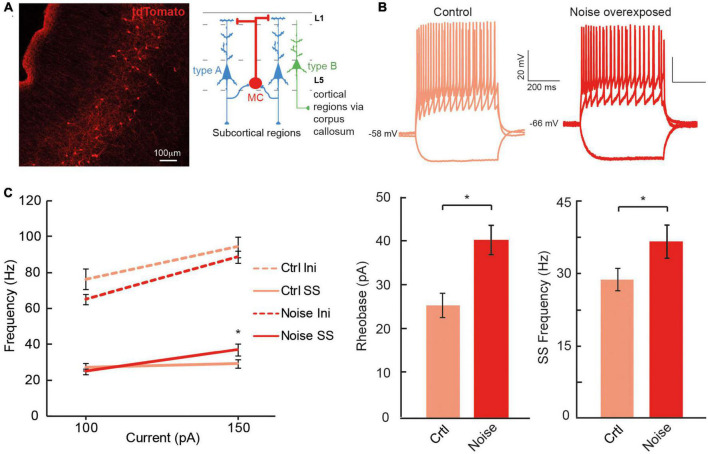
Chrna2 + MCs of the A1 increase steady state firing frequency after a noise overexposure. **(A)** Representative fluorescent image of Chrna2-Martinotti cells (red) in layer 5/6 of the A1. Inset shows the schematic connectivity of type A PC, type B PC and MCs in the A1. **(B)** Representative current clamp traces from MCs from control (left) and noise-overexposed (right) mice in response to –50, 50, and 150 pA. **(C)** Line plot showing a significantly higher steady state firing frequency after a noise trauma in response to 150 pA current injection (left). Bar graphs showing a significantly larger rheobase current (center) and steady state firing frequency (right) in MCs that have been exposed to noise compared to control mice. Error bars–s.e.m., Student’s *t*-test, two tailed, and equal variances, *denotes *p* < 0.05.

The dual response of type A and type BPC action potentials made us question whether loud noise exposure could also modify Chrna2-Martinotti cells (MCs), as L5/6 MCs have been shown to be specifically inhibiting type A PCs of the auditory cortex, and generating synchronous firing between type A PCs, while minimally affecting type B PCs (Hilscher et al., 2017; Figure 6A). We examined MCs membrane properties from transgenic c57BL/6 mice (cre expressed by the Chrna2-cre promoter) crossed with tdTomato-lox mice to generate red fluorescent protein in chrna2 positive cells (Leão et al., 2012; Hilscher et al., 2017, 2019) that were either submitted to loud noise-exposure 1 week prior to recordings or control age matched littermates, not noise-exposed. We found the resting membrane potential to be more hyperpolarized in Chrna2 + MCs from noise exposed mice compared to MCs from control Chrna2-cre/tdTomato-lox mice (ctr MC: −57.8 ± 1.3 mV, *n* = 40 vs. noise MC: −63.3 ± 1.6 mV, *n* = 19, *p* = 0.050) (Figure 6B and Table 3), no difference in average input resistance was found (ctr MC: 383.8 ± 25.8 MΩ, *n* = 32 vs. noise MC: 356.4 ± 37.7 MΩ, *n* = 19, *p* = 0.835) but an increase in current needed to generate an action potential (*rheobase:* ctr MC: 25.6 ± 2.8 pA vs. noise MC: 40.5 ± 3.4 pA, *p* = 0.013). Furthermore, an increased average steady state frequency (ctr MC: 29.2 ± 2.4 Hz vs. noise MC: 37.3 ± 3.5 Hz, *p* = 6.3 × 10^–5^) was seen in MCs from noise exposed mice in response to a 150 pA current step (Figure 6C). Other electrophysiological properties of Chrna2-Martinotti cells were not affected by the noise exposure (Table 3). This shows that MCs are more hyperpolarized at rest, and require more positive current to generate an action potential, but in response to higher (+100 pA) positive steps, the MCs from 1 week previously noise-exposed mice can fire at higher frequency compared to control mice.

**TABLE 3:** Membrane properties of L5 Chrna2 + Martinotti cells in the A1 of slices from control (*n* = 40) mice and mice with prior (1 week) noise exposure (*n* = 19).

	Control	Noise exposed	*p*-value
V_rest_ (mV)	-57.8 ± 1.3	-63.3 ± 1.6	0.037*
R_inp_ (MΩ)	383.8 ± 25.8	356.4 ± 37.7	0.835
Rheobase (pA)	25.6 ± 2.8	40.5 ± 3.4	0.012*
ΔSag (mV)	1.5 ± 0.5	3.3 ± 0.75	0.008*
ΔADP (mV)	8.5 ± 1.2	6.5 ± 1.3	0.631
ΔAHP (mV)	1.7 ± 0.5	1.0 ± 0.6	0.097
AP_thres_ (mV)	-40.5 ± 1.3	-41.0 ± 1.3	0.221
f_ini_ at 100 pA (Hz)	33.2 ± 3.8	63.7 ± 2.5	8.5 × 10^–5^*
f_ss_ at 100 pA (Hz)	17.7 ± 1.4	25.0 ± 2.0	*6.3* × 10^–5^*

Vrest, resting membrane potential; R_inp, input resistance; Rheobase, minimal current to cause an action potential; Sag, hyperpolarization sag; ADP, afterdepolarization; AHP, afterhyperpolarization; AP_thres, action potential threshold; f, firing frequency; ini, initial; ss, steady state; Student’s *t*-Test, two-tailed, and equal variance. Data show standard error of the mean (s.e.m). *p ≤ 0.05*.

## Discussion

This study shows that noise-exposure has opposite effects on *in vitro* firing frequency of two main types of L5 PCs of the primary auditory cortex of mice. Type A and B PCs are broad classes of PCs that differ in morphology and response to hyperpolarization. As it has been shown that subcortical/corticofugal projecting L5 PCs show a thick-tufted dendritic tree and prominent hyperpolarization-activated current (Ih) (i.e., type A PCs), while contralateral/callosal-projecting PCs show thin-tufted dendrites and have less Ih (i.e., type B) (Oswald et al., 2013; Lee et al., 2014), we used electrophysiological criteria for type A and type B L5 PCs (Lee et al., 2014; Hilscher et al., 2017). Thereby, the presence of h-current, generating afterdepolarization, together with afterhyperpolarization values, were sufficient to identify two broad PC classes. This study did not find any effect of noise exposure on h-current (as indicated by sag size) (Leao et al., 2006). Our previous voltage clamp data suggest that noise exposure may affect outward currents that control firing frequency (Leão et al., 2010). For example, 1 h of sound stimulation (4–12 kHz chirps, 75 dBSPL, 1 h) quickly increases high-threshold voltage dependent potassium channel (Kv3.1b) expression (responsible for sustaining high frequency firing) in brainstem auditory neurons (Leão et al., 2010). Future studies should aim to investigate how specific outward currents are affected by noise trauma, especially as potassium channel modulators may be used in tinnitus treatment (Sun et al., 2015; Henton and Tzounopoulos, 2021). Here we do not infer that mice have acute tinnitus, as no such tests were performed. Instead, we merely consider the persistent effect (after 1 week) of a session of loud noise exposure on single L5 A1 neurons.

To separate noise-exposure effects from maturation of membrane properties, such as lowering of input resistance and hyperpolarizing the AP threshold (Kroon et al., 2019), we opted to record neurons from 5–8 weeks old (P38-52) mice, similar to Gee et al. (2012) and Lee et al. (2014), as our initial experiments showed that the resting membrane potential decreased with age for type B PCs, but not type A PCs after the third week of age. Our data supports the work of Joshi and others in which mice aged P24-32 showed corticocollicular neurons from A1 to have an average resting membrane potential of −66 mV and cortico-callosal PCs a resting of −71 mV (Joshi et al., 2015), (our results for mature cells: type A −66 mV and type B −72 mV). Here we found resting membrane potential of type A and B cells to be indistinguishable in animals younger than P24 (young type B = −64 mV). Still, resting membrane potential is not reliable as an only indicator of PC subtype from animals >5 weeks of age, but emerged when averaging values following the type A and type B criteria. We further confirmed that the lack of distinct sag, ADP and AHP for type B PCs does not appear related to the age/resting potential.

Our study has several limitations, for example, not all neurons of the A1 respond to the noise overexposure applied due to limitations of the speaker frequency range. Still, by patching a random sample of type A and type B PCs we could identify differences in firing frequencies, supporting that the effects are strong enough to emerge and the principal component analysis of the variability of the sample supports our findings. In general, principal components are linear combinations of original variables and their meaning rises from directions (negative or positive) and magnitude of corresponding coefficients that generate each PrC. In this work, the first PrC can be regarded as an inertia of the PC to fire action potentials, due to higher values of rheobase, initial ISI, AP threshold and timing of first AP, all leading to higher values of PrC1. In the same way, lower values of initial and steady state frequency and resting potential also generate higher PrC1 values. Next, PrC2 may be interpreted as cells with higher values of steady-state frequency, f-I gain for steady state frequency, rheobase, initial frequency and f-I gain of initial frequency. On the other hand, cells with higher values of absolute sag, ADP and AHP and resting potential exhibit lower values of PrC2. Lastly, PrC3 may reflect firing of an action potential as the PrC3 is dominated by the values of AP threshold and input resistance. Visualizing the first three PrC of type A and type B L5 cells from control and noise exposed mice showed how the different cell types move further away from each other following a previous noise exposure, validating that the noise exposure has a persistent effect on L5 PC membrane properties, but effects are not the same for type A and type B PCs.

**FIGURE 7:**
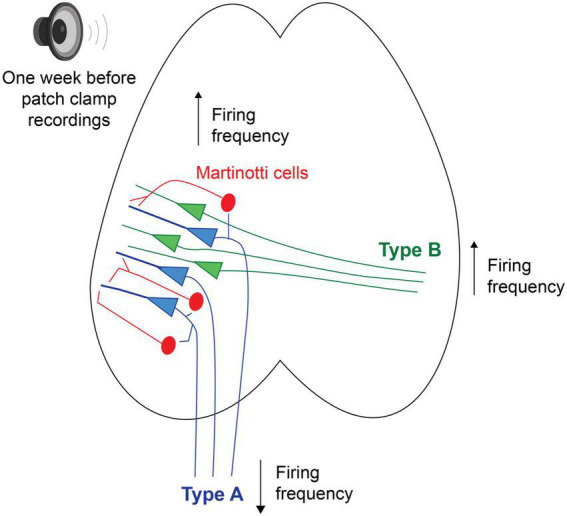
Schematic putative model of alterations in firing properties, 1 week following a session of noise overexposure, speculating that L5 type A PCs could receive increased inhibition from nearby MCs leading to decreased firing frequency, while L5 type B PCs showed increased firing frequency following noise overexposure.

Future studies using tracers and filling patched neurons with biotin will allow for a more detailed description of what anatomical structures and cell morphologies are more sensitive to loud noise. Asokan et al. (2018) has, for example, shown that corticofugal projecting L5 PC of the auditory cortex, with the main target of the ipsilateral inferior colliculus, also innervate the striatum, amygdala and the medial geniculate body. Furthermore they showed corticocollicular neurons to increase firing frequency gain for up to 2 weeks after a noise trauma, where instead auditory brainstem responses showed the wave 1 (cochlear nerve response) to decreased response gain (Asokan et al., 2018). Here our data show the opposite trend of type A PCs (possibly corticofugal projecting, Figure 7) showing a lower average steady state firing frequency 1 week after noise exposure. Still, Asokan et al. used a slow indicator for calcium imaging (GCaMP6s) to record increased response gain following noise exposure, which might capture a stronger synchrony of firing among corticocollicular neurons (i.e., type A PCs rather than reflect firing frequency). In addition, brief noise overexposure has been shown to generate reorganization of excitatory and inhibitory inputs onto inferior colliculus neurons and gap detection deficits in mice 7 days after a noise trauma (Sturm et al., 2017). It is known that noise overexposure can cause a multitude of circuit alterations (Shore and Wu, 2019) and here we also show that Chrna2-MCs show higher firing frequency in *in vitro* slices from noise exposed mice. We have previously shown that Chrna2-MCs activity in short bursts of 15 Hz can generate synchronous firing of type A PCs (Hilscher et al., 2017). Thereby we speculate that increased activity of MCs after noise exposure could generate a stronger type A output through increased synchrony to the descending auditory system, in line with increased corticocollicular response gain for several weeks after noise overexposure (Asokan et al., 2018). In addition, this could also indicate that MCs are more likely to inhibit L5 type A PCs and thereby contribute to the decrease in firing frequency of type A PCs seen here *in vitro*, as Chrna2-MCs provide distal dendrite inhibition to nearby (type A) PCs (Hilscher et al., 2017; Nigro et al., 2018; Figure 7). Still, it is necessary to further confirm these results exclusively in Chrna2-cre/tdTomato-lox animals to draw any specific conclusions on circuit modulation by noise overexposure.

It is also important to further investigate Chrna2-MCs as there might be additional subtypes of these interneurons, for example, Chrna2 positive interneurons of the striatum were recently shown to exhibit 3 subtypes (Tokarska and Silberberg, 2022). Still, Chrna2+ neurons of the hippocampus specifically labels *oriens-lacunosum/moleculare* (OLM) cells (Leão et al., 2012), however, we have shown that Chrna2+ OLM cells differ in h-current magnitude across the dorso-ventral axis of the hippocampal CA1 region (Hilscher et al., 2019). Here our criteria was to patch tomato expressing neurons of the A1 and quantify basic firing properties. A larger sample of Chrna2+ MCs may allow for further subdivision of membrane properties and cell morphology (Nigro et al., 2018) since the genetic profiles of Chrna2+ MCs show several possible subclassifications based on clustering of transcriptomic data (Allen brain Atlas, Transcriptomics Explorer).^1^

It is generally accepted that loud noise exposure can lead to noise-induced tinnitus by cochlear injury triggering peripheral deafferentation and adaptive changes in ascending auditory pathways (Elgoyhen et al., 2015). However, both human and animal studies have shown tinnitus without hearing loss (Langers et al., 2012; Longenecker and Galazyuk, 2016), but few studies separate the two conditions. Loud noise that does not significantly change hearing thresholds, but can generate acute tinnitus (Winne et al., 2020; Malfatti et al., 2022), similar to as used in this study, shows that PCs in the A1 still can alter input/output function following loud noise exposure. The increased firing frequency observed in type B PCs might indicate increased interhemispheric activity, as type B neurons are likely to project cortico-cortically (Lee et al., 2014; Joshi et al., 2015). Thereby, we speculate that even in the absence of underlying cochlear pathology such as hearing loss, plastic alterations in higher auditory areas could still sustain tinnitus by general increased interhemispheric activity. Increase in auditory cortex activity has been observed 1 day after loud noise exposure in mice with normal audiograms using resting-state functional magnetic resonance imaging (fMRI), but 28 days later the auditory cortex activity was instead decrease while limbic structures such as the amygdala showed increased activity (Qu et al., 2019). This shows the dynamic modulation of higher areas in response to loud noise, and that limbic structures are also part of tinnitus pathophysiology (Leaver et al., 2011). Thereby mapping of connectivity of subtypes of A1 L5 neurons projecting to limbic structures (Asokan et al., 2018), or their connectivity with areas regulating limbic structures, such as the basal forebrain cholinergic system (Joshi et al., 2016), becomes crucial to further extend our understanding of noise-induced tinnitus mechanisms.

In summary, we report that loud noise exposure can cause distinct effects on type A and B L5 PCs and inhibitory MCs of the mouse A1 one week after a noise exposure. Principal component analysis of membrane properties of type A and type B L5 PCs from >5 weeks old mice show parameters to spread in the opposite direction between the two subtypes of L5 PCs from noise-exposed mice compared to control mice. Whether this is due to increased activity of L5 MCs remains to be further elucidated, but together this study illustrates the moldability of membrane properties of A1 L5 neurons after exposure to loud noise.

## Data availability statement

The raw data supporting the conclusions of this article will be made available by the authors, without undue reservation.

## Ethics statement

This animal study was reviewed and approved by the Ethics Committee for the Use of Animals of Federal University of Rio Grande do Norte (CEUA/UFRN).

## Author contributions

IN: investigation, data curation, visualization, and writing—original draft preparation. TL: formal analysis and writing—original draft preparation. TM: software and validation. KL: conceptualization, methodology, and writing—review and editing. All authors contributed to the article and approved the submitted version.

## Footnotes

1
https://celltypes.brain-map.org
